# The Gate Theory of Pain Revisited: Modeling Different Pain Conditions with a Parsimonious Neurocomputational Model

**DOI:** 10.1155/2016/4131395

**Published:** 2015-12-27

**Authors:** Francisco Javier Ropero Peláez, Shirley Taniguchi

**Affiliations:** ^1^Center of Mathematics, Computation and Cognition, Universidade Federal do ABC, 09210-180 Santo André, SP, Brazil; ^2^Basic Sciences, Albert Einstein Hospital, 05521-200 Sao Paulo, SP, Brazil

## Abstract

The gate control theory of pain proposed by Melzack and Wall in 1965 is revisited through two mechanisms of neuronal regulation: NMDA synaptic plasticity and intrinsic plasticity. The Melzack and Wall circuit was slightly modified by using strictly excitatory nociceptive afferents (in the original arrangement, nociceptive afferents were considered excitatory when they project to central transmission neurons and inhibitory when projecting to substantia gelatinosa). The results of our neurocomputational model are consistent with biological ones in that nociceptive signals are blocked on their way to the brain every time a tactile stimulus is given at the same locus where the pain was produced. In the computational model, the whole set of parameters, independently of their initialization, always converge to the correct values to allow the correct computation of the circuit. To test the model, other painful conditions were analyzed: phantom limb pain, wind-up and wind-down pain, breakthrough pain, and demyelinating syndromes like Guillain-Barré and multiple sclerosis.

## 1. Introduction

The gate control theory of pain developed by Melzack and Wall in 1965 [1] proposes that tiny neural networks distributed along the dorsal horn of the spinal cord are responsible for relieving the pain in a specific body location when an intense tactile stimulation is applied at the same place. We experience this phenomenon in our daily life when rubbing the spot where an injury has just occurred.

**Figure 1:**
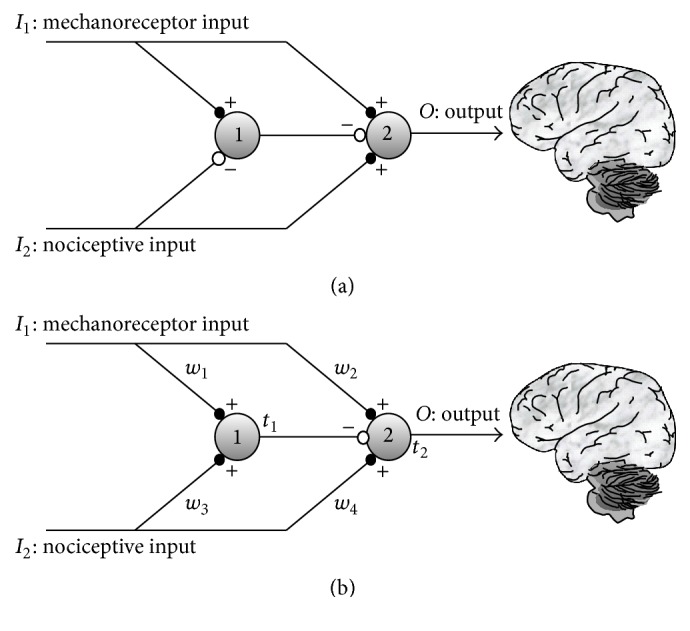
(a) The gate control mechanism proposed by Melzack and Wall in 1965. Both nociceptive and mechanoreceptors signals are projected to neurons in the substantia gelatinosa, represented by neuron 1, and towards the first central transmission neurons, represented by neuron 2. Mechanoreceptor signals are more intense (higher transmission rate) than nociceptive signals. Nociceptive signals inhibit neuron 1 (white dotted connection in figure) and, at the same time, produce excitation on neuron 2. (b) Current proposal: all nociceptive and mechanoreceptor axon terminals are excitatory. Synaptic weights (*w*_*i*_) change according to NMDA plasticity. Firing thresholds, *t*_1_ and *t*_2_, of neurons 1 and 2 also vary according to intrinsic plasticity.

According to them, axons of first order afferent nociceptors and low-threshold afferent mechanoreceptors converge to the same neurons in the substantia gelatinosa (SG) in the dorsal horn of the spinal cord, where inhibitory interneurons block nociceptive signals on their way to the brain. Since mechanoreceptors are low-threshold and their axons are myelinated, they produce high-rate action potentials. In contrast, nociceptive stimuli are less intense (in the sense of transmission rate) because they are transmitted through nonmyelinated axons.  Figure 1(a) shows the neural arrangement proposed by Melzack and Wall.

According to Wall, the gate theory of pain is not a final version so that its details might be discussed and improved.

“That a gate control exists is no longer open to doubt but its functional role and its detailed mechanism remain open for speculation and for experiment” [2].

Other authors (see [3, 4]) detected some flaws in the theory emphasizing the necessity of reviewing the gate control theory.

One controversial detail of the model (see Figure  4 in [1]) is that afferent nociceptors produce an excitatory stimulus on first central transmission (CT) neurons and, simultaneously, an inhibitory stimulus on neurons in the SG. This fact seems to contradict the idea that axon terminals of excitatory neurons are all excitatory, and axon terminals of inhibitory neurons are all inhibitory. This neuron's specialization is due to the absence of any mechanism in axons for guiding excitatory neurotransmitters from their origin at the neuron's soma to some specific axon terminals, while diverting inhibitory neurotransmitters to other terminals. By considering this, we have decided to test whether it is possible to obtain the normal operation of the gate with only excitatory synapses from nociceptive neurons. Note that in this case the gate circuit (see Figure 1(b)) is completely symmetrical: the same type of arrangement is present in both the upper and the lower halves of the circuit. The only difference between these two halves is the type of signal arriving from mechanoreceptors (in the upper half of Figure 1(b)) that is more intense than the signal from nociceptors (in the lower half of Figure 1(b)) arrangement.

Considering these ideas, we implemented a computational model of the neural circuit shown in Figure 1(b) with only excitatory connections from nociceptive and mechanoreceptors inputs. We have tested the operation of the model with and without the property of firing threshold adaptation also called intrinsic plasticity [5–7] and with and without NMDA plasticity [8–10] in their synaptic inputs. For a review of plasticity in pain, see Woolf and Salter (2000) [11] and Todd (2010) [12]. We did not model plasticity in the inhibitory synapses located at the axon terminals of SG neurons because plasticity is usually absent in axon terminals of inhibitory neurons although several types of inhibitory plasticity have been recently described in the literature [13]. As it will be shown in the following sections, the model only operates similarly to the gate when a standard type of stimulation is applied and when both kinds of plasticity are taken into account: synaptic plasticity that modifies the synaptic weights (synaptic efficiencies) and intrinsic plasticity (adaptability of the firing threshold). We consider that a type of stimulation is “standard” or “conventional” when, in first place, the four binary combinations of inputs *I*_1_ *I*_2_ (00, 01, 10, and 11) are sequentially or randomly presented to the gate circuit (note that “1” indicates presence of stimulus and “0” absence of stimulus). Besides, the standard type of stimulation should have another feature: mechanoreceptor stimuli should be more intense than the nociceptive stimuli. With this kind of “standard stimuli” and considering synaptic and intrinsic plasticity, synaptic weights and neurons' thresholds converge to very specific final stability values in which the normal behavior of the gate takes place. Once parameters are stabilized, the conventional behavior of the gate circuit is obtained; that is to say, pain is blocked in the gate when concomitant sensory and nociceptive stimuli are applied.

We are also going to demonstrate that when sensory and mechanoreceptor stimulation are not the standard ones, the final parameters' setpoint can be different and an anomalous pain condition can be produced. For example, in the case of demyelination of mechanoreceptor axons in multiple sclerosis [14] or in the Guillain-Barré syndrome [15], sensory and nociceptive stimuli can be similar in intensity so that, due to the symmetry of the circuit, the gate final setup can treat somatosensory stimuli as nociceptive, thereby relaying a pain sensation to the brain (CT neurons activation) in the presence of touch alone (mechanical allodynia). Another example of nonstandard combination of inputs to the gate is phantom pain sensed after a limb amputation in a specific spot of the nonexistent limb. Phantom pain spontaneously appears without stimulation. As it will be shown, such situation occurs because the final setup of parameters allows null stimuli to produce a CT neuron activation that is transmitted as a pain signal to the brain. Wind-up pain and wind-down pain will also be studied showing that stimuli intensity is determinant in the adjustment of gate circuit parameters. Finally, we study the case of breakthrough pain. It corresponds to a situation in which gate parameters cross an unstable equilibrium point before reaching the final equilibrium setup where a situation of intense pain prevails.

## 2. Methods

### 2.1. Configuration of Training Epochs

As it has been suggested in the Introduction, a critical aspect to reach a certain pain condition is the way sensory and nociceptive inputs are combined when they are input to the gate circuit. The four possible types of inputs combination are shown in Table 1(a) in which bit = 1 means presence and bit = 0 absence of input.

In neural networks literature, a training epoch is a set of input patterns that is repeatedly presented to a neural network. In our case, an epoch is the set of the four possible inputs combinations shown in Table 1(a) but with graded sensory and nociceptive inputs as in Table 1(b). In this case, Table 1(b) shows a specific type of epoch used to train the network in a standard way. Numerical values correspond to input neuron's firing probabilities and are arbitrarily selected to represent a stimulation regime. For example, in a standard stimulation regime, the firing probability from sensory (mechanoreceptor) inputs is higher than the firing probability of nociceptive ones.

Human subjects categorize the intensity of stimuli using verbal expressions like very intense, intense, medium, low, and so forth. It is reasonable to think that an intense stimulus produces a higher firing probability in sensory/nociceptive receptors than a medium stimulus. Given that, up to now, there is not a universal criterion for associating a verbal expression with a firing probability value; we have defined a preliminary scale for the qualitative purposes of the present research.

For translating the verbal expressions indicating the intensity of sensory/nociceptive inputs into firing probabilities, we arbitrarily elaborate Table 2.

With this kind of table, it is possible to translate into firing probabilities, a sentence like the following.

“During a standard kind of stimulation, sensory inputs are of medium to intense intensity being nociceptive inputs weak. In the case of a demyelinating syndrome, sensory inputs become weak. Dysesthesia might take place with a subsequent regime of stimulation with medium nociceptive inputs.”

We will use this verbal description in Section 3.2.2 for studying demyelinating syndrome pain.

### 2.2. Neuron Model

Neurons used in the model belong to the very simplified rate-code neuron type: their outputs, *O*, representing their firing frequencies. In the rate-code output model, the probabilities of an action potential in the presynaptic and postsynaptic neurons are, respectively, written as *I* and *O*. The synaptic weight *w* relates *I* and the excitatory postsynaptic potential, *E*, at synapse *j*:

(1) $$\begin{array}{c} E_{j}=w_{j}I_{j}. \end{array}$$

The postsynaptic action potential probability is given by a nonlinear (sigmoidal) function of neuron's net input: *O* = *f*(net). Such net input, Net, is obtained after summing the postsynaptic potentials of all synapses:

(2) $$\begin{array}{c} \text{net}=\overset{n}{\underset{j=1}{\sum }}E_{j}=\overset{n}{\underset{j=1}{\sum }}w_{j}I_{j}. \end{array}$$

The sigmoidal function of neuron's activation yields the probability of an output action potential and is given in our simulations by

(3) $$\begin{array}{c} O=\frac{1}{1+e^{-k\left(net+0.5-2s\right)}}, \end{array}$$

where *s* is a parameter that contributes to modeling the neuron's firing threshold, *t*, where *t* = 2*s* − 0.5. *k* is a curve-compressing factor that was set to 50 for modeling a steep slope of the sigmoid function. The range of *s* is 0 < *s* < 1. For *s* = 0, the sigmoid is completely shifted leftwards so that, for net = 0, *O* = 1. In the case *s* = 1, the sigmoid is completely shifted rightwards so that, for net = 1, the output value of the sigmoid is *O* = 0.

### 2.3. Adjustable Properties: Synaptic and Intrinsic Plasticity

We have demonstrated elsewhere [16] that NMDA plasticity (that, according to Woolf and Thompson [9] is present at SG neuron synapses) can be modeled through a probabilistic rule. Since we are utilizing rate-code neurons with outputs indicating a probability and not real binary outputs, we need to obtain at least a fictitious binary output for calculating the weight value, *w*, of NMDA synapses. The fictitious output of the presynaptic neuron (bit = 1 or bit = 0) is denoted by *i* (lowercase) and the postsynaptic fictitious output (bit = 1 or bit = 0) is denoted by *o*. Binary values *i* and *o* are randomly generated with probabilities *I* and *O*, respectively. With these binary values, the correlation among synaptic inputs and outputs is calculated by means of the probabilistic version of the so-called presynaptic rule, which is the conditional probability:

(4) $$\begin{array}{c} w=P\left(\;\frac{o}{i}\;\right)=\frac{n\left(o\cap i\right)}{n\left(i\right)}, \end{array}$$

where *n*() in the numerator counts the number of concurrent presynaptic and postsynaptic unitary binary outputs.

The apparent shortcoming of this probabilistic synaptic weight modeling is that the higher the weight the more correlated the presynaptic and postsynaptic neurons so that weights become higher. At the end, it seems that weight values have no other possibility rather than growing until they saturate, that is, turn into one.

Fortunately, neurons have another property that contributes to moderating the tendency of synaptic weights to increase until saturation. This property, called intrinsic plasticity [5, 6], either increments or decrements the neurons firing threshold so that the neuron is, respectively, less prone or more prone to fire in the future. Neurons in spinal cord laminae III–VI, that is, in deep dorsal horn, express intrinsic plasticity (see a comprehensive review in Sandkühler, 2009 [7]).

**Figure 2:**
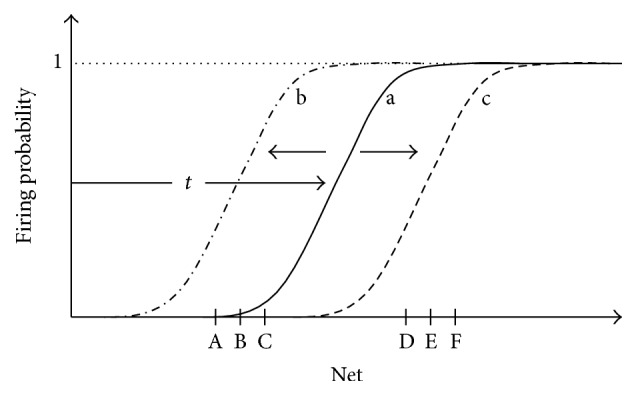
Intrinsic plasticity is the property of real neurons that allows the neuron's sigmoidal activation function to shift either leftwards or rightwards, so that the sigmoid is placed over intervals corresponding to the average net input of the neuron. (a) Initial position of the sigmoidal activation function. (b) If the values of net inputs of the neuron are low (as in case of inputs A, B, and C), the activation function shifts leftwards. (c) If net input values are high (as in D, E, and F), the sigmoid gradually shifts rightwards.

Rigorously, there is not a clear cut-edge defining a threshold that changes the neuron firing probability from zero to one. Instead of this, the transition is governed by the sigmoid function presented in (3) that is also depicted in Figure 2, in which the firing probability makes a gradual transition from zero to one. For us, the firing threshold will be defined as the value of net input that makes the neuron fire with probability equal to 0.5.

We have demonstrated elsewhere [16] that synaptic weights increment can be counterbalanced by the dynamic adjustment of the shift of the sigmoidal function so that the more the synaptic weights (and accordingly the net input value) grow, the more the shift grows. Thus, the steepest slope of the sigmoid tends to be placed over the average net input of the neuron (see Figure 2). Such dynamic adjustment makes synaptic weights stop increasing and stabilize in specific values.

The following equation modeling intrinsic plasticity calculates the shift parameter of the activation function, *s*, at time *t* in terms of the shift parameter and output probability of the neuron at time *t* − 1:

(5) $$\begin{array}{c} s_{t}=\frac{\upsilon \cdot O_{t-1}+s_{t-1}}{\upsilon +1}, \end{array}$$

where *υ* is a small arbitrary factor that adjusts the shifting rate of the activation function.

As previously mentioned, parameter *s* allows the calculation of firing threshold, *t*, which is

(6) $$\begin{array}{c} t=2s-0.5. \end{array}$$

Considering that the range of variable “net” after weight stabilization is in the [0,1] interval, the arbitrary election of this equation for modeling threshold, *t*, allows to have the sigmoid completely shifted rightwards with *s* = 1 and completely shifted to the left for *s* = 0.

In this paper, we show that the dynamic interactions between synaptic and intrinsic plasticity are the factors that allow the stabilization of parameters in the gate circuit. Once parameters are stabilized under either standard or altered modes of operation, they give rise to either normal or altered pain sensations.

### 2.4. Some Notes regarding Units, Scales, and Iterations

In this research, we use a phenomenological type of neuron modelling. We take into account that, at least in mammals, rate coding is the way neurons communicate with one another. In gate circuit models, the neuron's output *O* is a rate (3) value. Such rate can be expressed in the form of a probability ranging from zero to one. When *O* = 1, it means that the neuron fires every time it is possible. Probabilities are dimensionless measurements because they are obtained from the quotient of equal type of magnitudes (see (4)). For this reason, in the graphs of this paper, unit specification does not appear in the vertical axes representing neuron's output, *O*.

Synaptic weights are also calculated as conditional probabilities and are, therefore, dimensionless. For this reason, graphs representing weights do not have unit specifications in their axes. In a similar way, variables that are obtained as a combination of dimensionless variables are also dimensionless like the net input, net, and the shift parameter, *s*. When these variables appear in a graph, they are devoid of unit specifications.

A comparison with binary bits might help in the understanding of these ideas. A binary bit is a dimensionless magnitude that is either 1 or 0. For computers to work, binary 1 is arbitrarily associated with a certain voltage level that depends on the available technology. In TTL (transistor-transistor logic) technology, binary 1 is arbitrarily associated with a 5 volts' voltage level and binary 0 with a 0 volts' level. In a similar way, the net input value, that is dimensionless, is associated in real neurons with specific postsynaptic voltage levels.

Regarding time comparisons, computers use “iterations” to perform their instructions. An iteration is typically a sequence of tasks in a programming loop. In our model, the four possible combinations of sensory/nociceptive inputs that integrate an epoch are processed inside an iteration. When each combination of sensory/nociceptive inputs feeds the gate circuit neurons, their net inputs and outputs are calculated, and their firing thresholds and synaptic weights are altered. According to this, we can say that the real counterpart of an iteration in our computer model would be a period of time in which all combinations of sensory/nociceptive stimulus are given to a subject with specific intensity degrees. In order to relate iterations, time windows, and evolution of patients with stimulation protocols, patterned experimental tests with real patients should be necessary.

## 3. Results

This section is devoted to analyzing the behavior of the gate circuit under standard (Section 3.1) and nonstandard (Section 3.2) types of stimulation.

In Section 3.1, we will show that the conventional gate operation is achieved once gate parameters (intrinsic and synaptic plasticity) stabilize after a dynamic transitory period. During this transitory period, a standard training epoch (depicted in Table 1(b)) is input to the gate, being the mechanoreceptor input higher than the nociceptor input. As it will be demonstrated, synaptic and intrinsic plasticity interact for allowing parameters stabilization and convergence because one type of plasticity counterbalances the other. Under such condition, the operation emerging from the circuit consists of pain only being elicited (CT neurons fire) when nociceptive signals are the only input to the circuit which is the conventional gate circuit operation.

At the end of Section 3.1, we will see that the adequate convergence of parameters does not take place when intrinsic plasticity or synaptic plasticity alone is taken into account. The interplay of both synaptic and intrinsic plasticity is necessary to create the conditions for the circuit to reach the setpoint that makes the circuit respond in the conventional way.

In Section 3.2, we are going to study other pain conditions that result from anomalous training epochs given to the network, that is, (a) when mechanoreceptor input is equal to or lower than nociceptive input as in demyelinating syndromes, (b) when inputs are absent as in phantom pain, (c) when continuous weak sensory inputs produce an abrupt increment of pain sensation (wind-up pain), (d) when a continuous intense nociceptive input produces a temporary analgesia (wind-down pain), and (e) when an intense pain generates a transitory wind-down episode followed by breakthrough pain.

### 3.1. Standard Pain Responses due to Standard Stimulation Regime

**Figure 3:**
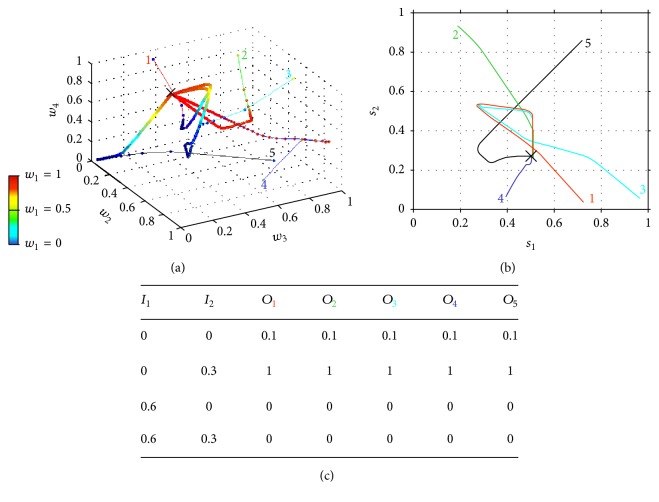
Evolution of gate circuit parameters when considering both intrinsic and synaptic plasticity. Five program simulations (5 thin colored lines) are depicted, starting with different initial weights and shifts. (a) Evolution of weights: each coordinate (*w*_2_, *w*_3_, *w*_4_) represents the set of synaptic weights in each iteration, with the color of the point representing the value of weight *w*_1_. Along iterations, all lines converge to the same coordinate (*w*_1_, *w*_2_, *w*_3_, *w*_4_) = (1, 0, 0.5, 0.5). (b) After 5000 iterations, the shift parameters of the activation function of the SG neuron (*s*_1_) and of the T neuron (*s*_2_) also converge to a certain point (0.5, 0.27). (c) With the final set of weights and shift parameters, the probability of a CT neuron's firing is given by the table being *I*_1_ the mechanoreceptor input probability and *I*_2_ the nociceptive input probability.

As previously mentioned, in this section, we will study the evolution of synaptic weights, *w*_*i*_, and shift parameters, *s*_*i*_, when a standard type of stimulation is input to the gate circuit. The model uses intrinsic plasticity in SG and CT neurons and NMDA synaptic plasticity in excitatory synapses. In order to allow the conventional gate operation, the interplay between intrinsic and synaptic plasticity is necessary for parameter stabilization and convergence. Equation (4) has been used for modeling weights modification and (5) was used to model the gradual shift of the activation function.  Figure 3 shows five trajectories of weights (Figure 3(a)) and shifts (Figure 3(b)) corresponding to five different simulations (five colored lines with the same color numbers), each of them starting with different shifts and weight values. As we have four weights *w*_1_, *w*_2_, *w*_3_, and *w*_4_ (see location of each of these weights in Figure 1(b)), a four-dimensional coordinate system would be necessary for representing their evolution along iterations. We have managed to represent the trajectory of the four weights along 5000 iterations in a three-dimensional coordinate system (see Figure 3(a)) by representing the value of *w*_1_ as a colored point over each trajectory. The color of each point corresponds to a color scale in which dark blue means *w*_1_ = 0 and red means *w*_1_ = 1. The values of *w*_2_, *w*_3_, and *w*_4_ are represented in a conventional three-dimensional system. All weight values are dimensionless and range from 0 to 1 because they are obtained from a conditional probability equation (4).

Notice that the five weight trajectories converge to the same coordinate: *w*_1_ = 1, *w*_2_ = 0, and *w*_3_ = *w*_4_ = 0.5. Shift trajectories (Figure 3(b)) also converge to the final shifts values *s*_1_ = 0.5 and *s*_2_ = 0.27 that, according to (6), correspond to the firing thresholds: *t*_1_ = 0.51 and *t*_2_ = 0.02. This final coordinate is marked with “*x*” in Figures 3(a) and 3(b).

**Figure 4:**
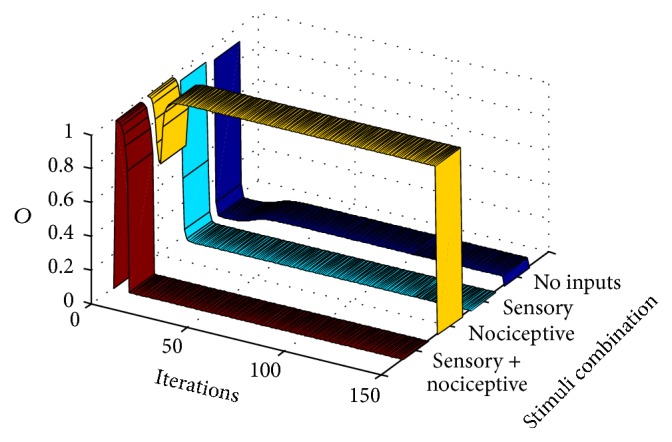
This graph shows the output, *O*, of the CT neuron in the gate circuit along computer iterations. It shows that the circuit quickly adapts, for eliciting standard gate outputs when standard pain and sensory signal are input to the circuit (a standard training epoch is given to the circuit in each iteration). Each ribbon represents the output of the circuit when a certain combination of inputs is introduced as input to the circuit. The dark blue ribbon yields the output (the CT neuron action potential probability) when no inputs are introduced. Cyan, yellow, and red ribbons yield the CT neuron output under conditions in which only sensory, nociceptive, or both inputs are, respectively, input to the gate circuit.

The table in Figure 3(c) is obtained at the final points of each colored trajectory. In the table's first row, the *i*th colored subindex of *O*_*i*_ refers to the same color trajectory. The table shows the output value, *O*_*i*_, of the CT neuron when applying a standard epoch to the circuit using the final parameters of each of the *i*th trajectories. In this case, the final parameters in the five trajectories are equal, so that the output when applying a standard epoch to each of the five circuit configurations is the same. In this case, the outputs correspond to the standard or conventional* modus operandi* of the gate circuit in which the CT neuron is only active when only the nociceptive input is active. The output probability that is equal to 0.1 when inputs *I*_1_ and *I*_2_ are equal to zero depends on the value of the sigmoidal function in zero. If a more realistic approximation of the activation function was used, in which *f*(0) = 0, the output probability would certainly yield zero. Graph of Figure 4 also represents the output *O* of the gate circuit (i.e., the output of the CT neuron) similarly to table in Figure 3(c). The difference between the table and Figure 4 is that the table shows the response of the circuit at the final setpoint marked with “*x*” in Figure 3(a). Figure 4, instead, shows the response of the circuit along iterations for each combination of inputs (in this case, in a standard epoch). For example, the dark blue ribbon shows the output of CT neuron when both inputs are null (*I*_1_ = 0 and *I*_2_ = 0) from the very first iteration until iteration 150. The cyan ribbon represents the response of the circuit along iterations when only a sensory signal is given (*I*_1_ = 0.6 and *I*_2_ = 0).

We see that under these conditions there is no CT neuron's output (there is no pain signal from the gate circuit when a sensory input alone is given). The yellow ribbon represents the output when only a nociceptive signal (*I*_1_ = 0 and *I*_2_ = 0.3) is input. In this case, the CT neuron's output quickly grows from the very first iterations so that a pain signal is only elicited by the gate circuit when a pure nociceptive input enters the gate. Finally, the flat dark red ribbon shows that no pain signal is elicited by the gate circuit along all iterations when both sensory and nociceptive input are simultaneously entering the circuit.

In summary, Figure 4 shows that the interplay between intrinsic and synaptic plasticity, together with the presentation of a standard type of input patterns, leads the gate circuit to the gate conventional* modus operandi*: when both sensory and nociceptive inputs are simultaneously input to the gate, CT neurons are silent. However, when nociceptive signals are input alone, they produce the CT neuron's output.

**Figure 5:**
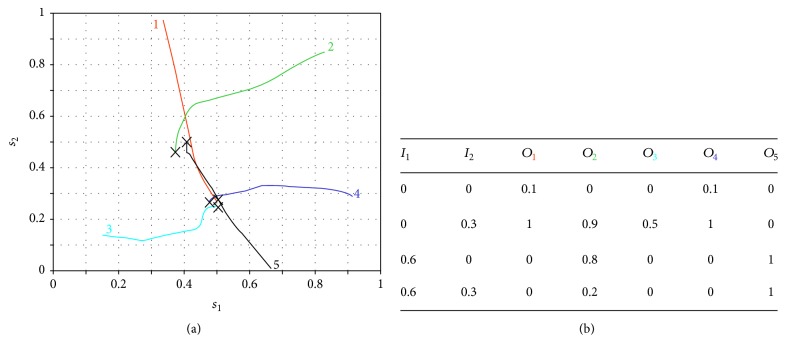
Evolution of gate circuit parameters with only intrinsic plasticity. (a) Evolution of the shift parameters of neurons 1 (SG) and 2 (CT) in five different simulations (different line colors). Crosses represent the values of shift parameters at the last iteration. (b) Truth table after each one of the simulations. Colors in the indexes refer to the same color curves in (a).

**Figure 6:**
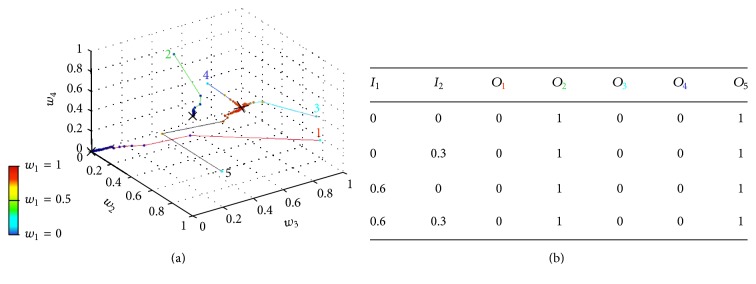
Evolution of gate circuit parameters with only synaptic plasticity. (a) Five computer simulations (five colors of narrow lines) representing synaptic weights evolution during 5000 iterations, once the shifts of activation functions are fixed. Because there are four weights and we have a 3D coordinates system, one of the coordinates, *w*_1_, is measured by a scale of colors ranging from 0 to 1. A cross indicates last iteration. (b) Truth table for each one of the simulations.

We performed other similar simulations, but only considering either intrinsic (Figure 5) or synaptic plasticity (Figure 6). As before, the five thin colored lines represent parameters evolution from five different initial conditions. It can be noticed that with only one type of plasticity there is no convergence of parameters and the gate circuit does not respond in a standard manner when a standard type of stimulation is applied.

### 3.2. Nonstandard Pain Responses due to Nonstandard Stimulation Regimes

In this section, we will show computer simulations that reveal how neuropathic pain evolves from a normal pain situation in terms of sensory/nociceptive stimulation. Our premise here is that the gate circuit is a kind of neural network that is trained (achieves different parameter configurations) depending on the type of external stimulation. For creating the initial standard conditions, the gate circuit is trained with a standard set of sensory/nociceptive inputs (as in previous sections) during the first 50 computer iterations. In this way, at iteration 50, the gate circuit is programmed as in Section 3.1. From iteration 51 ahead, the circuit is exposed to an abnormal training epoch (abnormal set of sensory/nociceptive patterns), which depends on the type of syndrome being modeled. In this paper, we model different syndromes: phantom pain, demyelinating pain syndromes like multiple scleroses or Guillain-Barre syndrome, breakthrough pain, wind-up pain, and wind-down pain.

#### 3.2.1. Phantom Pain Simulation

Let us start with the so called “*phantom pain*” [17] appearing in a nonexisting limb after amputation.

**Figure 7:**
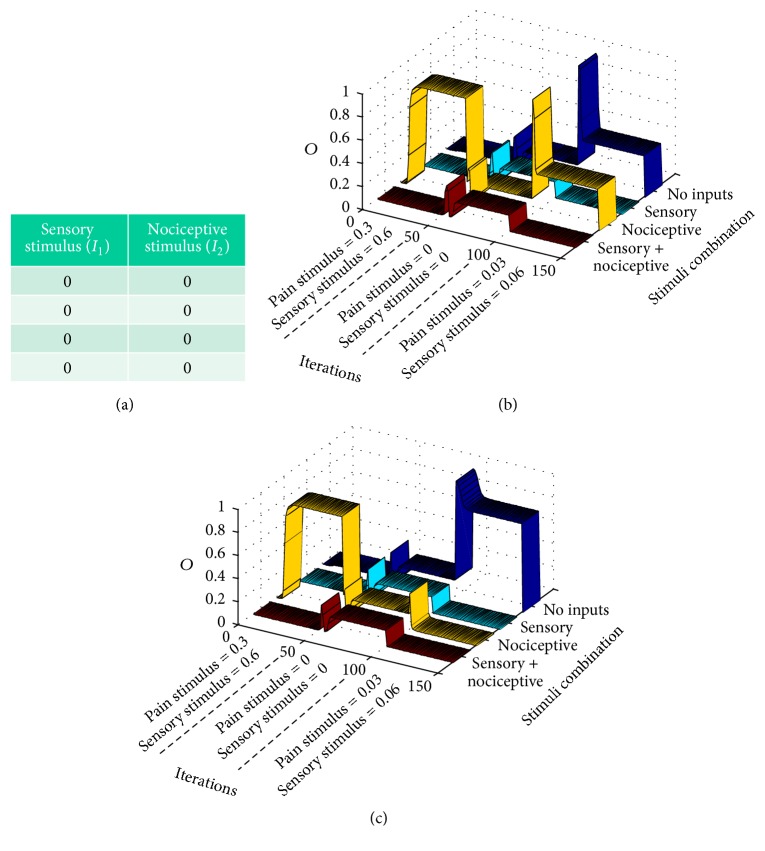
Modeling phantom limb pain: after presenting a standard training epoch to the gate model during 50 iterations, (a) a null training epoch is input to the gate along 50 more iterations in order to model the period after amputation. (b and c) After 100 iterations, very weak input signals are input to the gate model. Graphs correspond to two stability points. Both graphs show that pain signals are emitted from CT neurons in a situation in which there are no inputs to the gate in a condition known as dysesthesia (blue ribbon). Graph (b) shows a situation of a setpoint in which weak nociceptive inputs elicit a pain signal from CT neurons (yellow ribbon).

As it has been mentioned, the gate circuit is initially set to behave in a standard way by inputting a standard training “epoch” during its first 50 iterations. Once the circuit settles down, we model the amputation by zeroing both sensory and nociceptive inputs along the following 50 iterations (see Figure 7(a)). From iteration 101 to iteration 150, very subtle sensory and nociceptive inputs enter the circuit, simulating abnormal action potentials fired by neuromas (formed from injured nerve endings at the stump site).  Figure 7(b) shows the CT neuron output, *O*, along the mentioned iterations for the different nociceptive and sensory combinations. We can see that, from iteration 101 ahead, two cases are possible depending on the stability point in which model parameters settle down. In graph (b), we notice that a CT neurons output (a pain signal) is produced when either a nociceptive input is present (yellow ribbon) or no inputs are present at all (dark blue ribbon). In graph (c), a pain sensation (CT neuron output) appears when there are no input signals at all (dark blue ribbon). Although the peripheric pain component due to neuromas is not difficult to accept, pain when no input at all is present at the gate is more difficult to understand. This case is consistent with clinical findings that demonstrate that phantom pain remains even when local anesthesia is applied to the stump. The general consensus trying to explain this last case is that phantom pain is a top-down phenomenon caused by maladaptive cortical plasticity. However, a recent article [18] has reopened the discussion regarding the peripheral versus central origin of phantom pain. The results of our computational model shows that the gate circuit cannot be understood anymore as a “gate” that allows/precludes nociceptive signals, but as a type of signal processor that either generates or does not generate a pain signal according to the input signals and to the gate internal parameters configuration. Instead of always mitigating pain, the gate circuit is also able to create pain (produce a CT output), even in the absence of sensorial and nociceptive stimuli. For a neuron to fire in the absence of stimuli, the only possibility is that it has incremented its excitability by lowering its firing threshold (according to intrinsic plasticity). In the case of our simulations, the CT neuron lowered its threshold to* t* = −0.05. The whole set of parameters for the case of Figure 7(c) is shown in Figure 12(b).

#### 3.2.2. Demyelinating Syndrome Simulation

Let us model a* demyelinating syndrome* like multiple scleroses or Guillain-Barré syndrome that appears after 50 iterations of a standard pain situation. This case is also an example on how we translate a verbal expression like the one presented at the end of Section 2.1 for developing a stimulation protocol.

**Figure 8:**
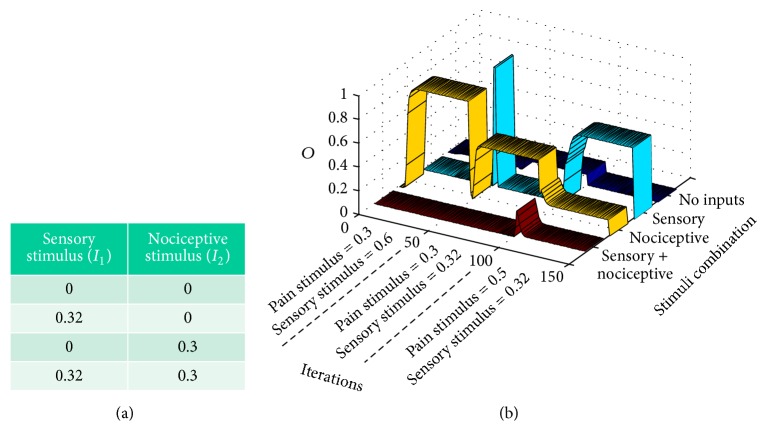
Modeling demyelinating syndromes: after 50 iterations with standard training epochs, signals from sensory receptors are weakened like in a demyelinating syndrome. Abnormal pain sensations only take place from iteration 100 with pain sensations when a sensory signal is input to the gate (dysesthesia).

In this case, the demyelinating syndrome makes mechanoreceptors convey weaker signals to the gate circuit. Table in Figure 8(a) shows the training epoch used from iteration 50 to iteration 100. Notice that sensory stimuli are weaker than in the standard training epoch, almost similar to stimuli from nociceptive nonmyelinated fibers. Here, we see that dysesthesia appears from iteration 101, at the onset of some concomitant event generating more intense nociceptive signals (in this case, a new training epoch was applied with nociceptive stimuli rising from 0.3 to 0.5). In these new conditions, pain sensations appear with sensorial stimuli (cyan ribbon).

Although gate parameters usually converge to an equilibrium point like the previously described, other equilibrium points are possible in demyelinating syndromes. This fact is in accordance with the literature that mentions different types of pain associated with demyelinating diseases like the Guillain-Barré syndrome (see [19]) and multiple scleroses (see [20]).

#### 3.2.3. Breakthrough Pain Simulation

Breakthrough pain [21] is defined as “a transitory exacerbation of pain experienced by the patient who has relatively stable and adequately controlled baseline (background) pain” [22].

**Figure 9:**
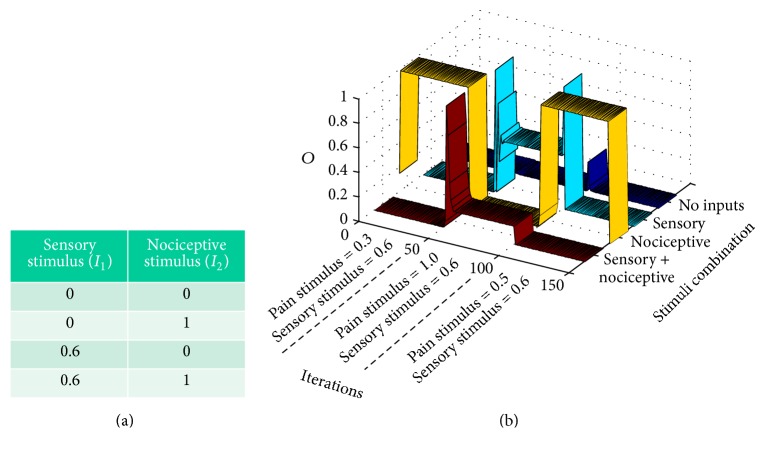
Modeling breakthrough pain: after 50 iterations with standard training epochs, an intense nociceptive stimulus is input to the gate. Initially, wind-down pain (yellow ribbon) takes place concomitantly with a mild episode of dysesthesia (cyan ribbon). After a period in which pain seems to be relieved, pain is again installed as in breakthrough pain.

In this case, after the preliminary standard setup during the first 50 iterations, the training epoch in Figure 9(a) is input to the gate circuit. After a few iterations, a wind-down phenomenon takes place in which nociceptive input signals (see yellow ribbon) produce decreasing pains sensations (CT neurons output). When pain sensations seem to be less intense, pain is triggered again when a moderate nociceptive stimulus is applied. Under the same circumstances, that is, intense nociceptive stimuli, other sequences are also possible. Nonstandard pain responses differ from the standard case in that in nonstandard cases there are usually secondary stability points in which pain responses are not so easy to predict.

#### 3.2.4. Wind-Down Pain

In this case, we will analyze wind-down pain which also was a pain response that appeared in previous case. Wind-down pain is usually experienced when intense aversive nociceptive stimuli are constantly applied. We performed the computational model of this case by letting the circuit settle under standard conditions during the first 50 iterations. From iterations 50 to 100, the unique stimulus applied was an intense *I*_2_ input in the nociceptive entrance.

**Figure 10:**
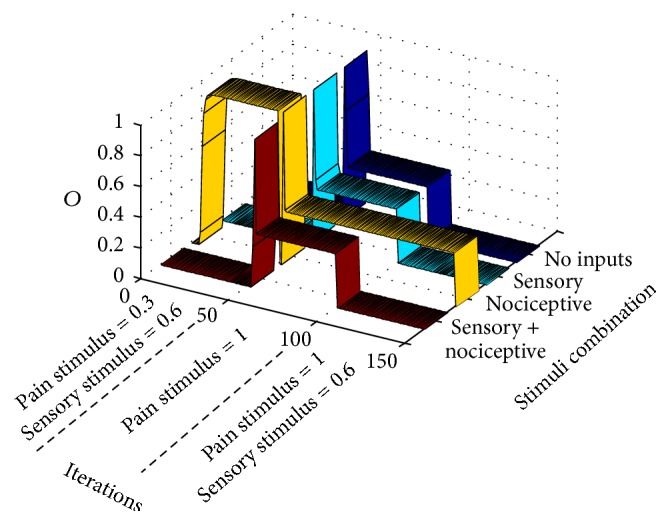
Modeling wind-down pain: after 50 iterations of standard training epochs, signals from nociceptive receptors become very intense. From iterations 50 to 100, the gate circuit, instead of receiving different stimuli like in previous cases, receives an intense nociceptive stimulus of value 1. For testing pain responses to other types of stimuli during the phase of intense nociceptive stimulation, all types of plasticity (synaptic and intrinsic) are blocked from iterations 100 to 150 (this procedure was not done in previous examples). As seen, after a long intense pain stimulation, the circuit becomes less responsive to all types of stimuli.

In order to better understand the response of the circuit to the four combinations of inputs without altering parameters' setup at the end of the intense nociceptive input presentation, we block synaptic and intrinsic plasticity from iterations 100 to 150 (this “parameters freezing” procedure was not performed in previous cases). In this way, we tested the circuit with all the combination of nociceptive and mechanoreceptor inputs. As seen at the final “tail” of colored ribbons of Figure 10, there is a reduced response to any of the combinations. In cancer pain, this wind-down component can be masked by the concomitant usage of analgesics like morphine [23], so that pain relief is erroneously thought to be due to the pharmacological treatment rather than being derived from neural plasticity dynamics.

#### 3.2.5. Wind-Up Pain

**Figure 11:**
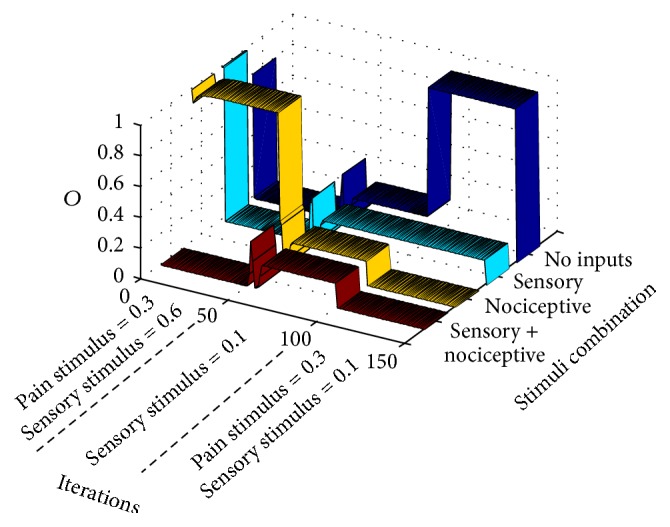
Modeling wind-up pain. After 50 iterations with standard training epochs, signals from mechanoreceptors become weak but repetitive, without any other type of stimulation. This situation is modeled from iterations 50 to 100 in which the gate circuit, instead of receiving different stimuli like in previous cases, only receives a repetitive weak sensory stimulus. For understanding pain responses to other types of stimuli during the phase of repetitive weak sensory stimulation, all types of plasticity (synaptic and intrinsic) are blocked from iterations 100 to 150 (as done in previous example). As can be seen, after a prolonged weak sensory stimulation, the circuit relays a pain output in the case of no stimulation (between periods of weak sensory stimulation).

Wind-up pain is the pain response elicited when a constant sensory weak stimulus is input through mechanoreceptors. The consequence of this apparently innocuous procedure is that, in the long run, an intense pain appears in the subject. We have simulated the conditions of wind-up pain from standard conditions (Figure 11) by initially letting the circuit settle in a stable point along the 50 initial iterations. From iterations 50 to 100, the only stimulus is a weak (0.1) mechanoreceptor one. To test the circuit response immediately after the stimulation procedure, avoiding altering circuit parameters, we block plasticity from iterations 100 to 150 and introduce the four mechanoreceptor/nociceptive stimuli combinations. As it can be seen, there is an intense pain when no input is applied to the circuit. In this case, the pain that is actually felt by the subject undertaking repetitive weak sensory stimulation seems to be a type of dysesthesia that possibly comes up between repetitive sensorial stimuli.

## 4. Discussion

One of the objectives of this work is to highlight the dependence of pain responses on gate circuit parameters (synaptic weight and firing threshold values) and on the relative contribution of afferent mechanoreceptors and nociceptors.

**Figure 12:**
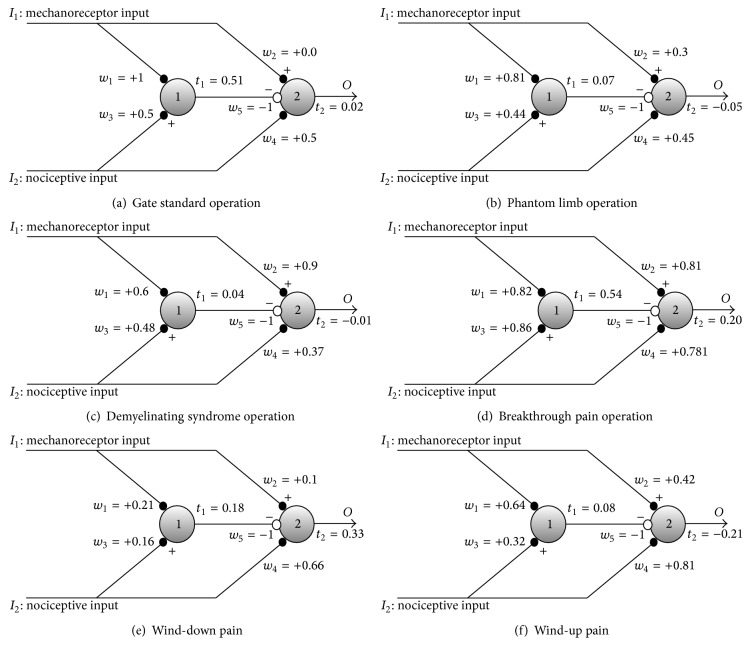
Synaptic weights and firing threshold values when the gate circuit arrives to stability for the different cases previously explained. When placing the different type of inputs in the gate circuit, neuron 2 response is according to the type of response expected in each case. In the case of graph (b), we considered the parameters of gate circuit that only produces phantom pain when no inputs at all enter the gate (see Figure 7(c)).

Usually afferent mechanoreceptors have myelinated axons generating more intense responses than nociceptors. At the same time, sensory and nociceptive stimuli are usually delivered to the central nervous system according to a stimulation protocol (epoch) that is similar to the standard one presented in Section 2.1. Under these standard conditions, the synaptic weights and firing thresholds of the gate circuit evolve until settling in a stable point that allows the conventional operation of the circuit (see synaptic weight values, *w*, and firing threshold values, *t*, in Figure 12(a)).

This conventional operation means that when a mechanoreceptor's input alone is input to the gate circuit or when mechanoreceptor and nociceptive inputs are both input to the circuit, no pain (CT neuron response) is relayed.  Table 3 helps in calculating the output of the circuit, *O*_2_, for each of the *I*_1_ *I*_2_ combinations of sensory/nociceptive inputs under standard conditions.

Table 3 uses the final weights and neuron's firing thresholds shown in Figure 12(a). For calculating the output of neuron 1, *O*_1_, the computer program applies the sigmoidal activation function of (3) to its net input (see (2)). As our sigmoid is very similar to a step function, it is also possible to obtain *O*_1_ by simply comparing Net_1_ with threshold *t*_1_. If the net input of neuron 1 is higher than its threshold, neuron 1 output is 1, which is almost the same result of applying the sigmoid to Net_1_. For calculating the final response of neuron 2, it is necessary to introduce the inhibitory contribution of SG neuron, as follows: Net_2_ = *w*_2_ *I*_1_ + *w*_4_ *I*_2_ − *O*_1_. The final output, *O*_2_, results from applying the sigmoidal function to Net_2_, that is roughly the same result as checking whether Net_2_ is higher than threshold *t*_2_, as explained.

A similar table might be elaborated for testing the response of the circuit after settling in a nonstandard set of parameters (see Figures 12(b), 12(c), 12(d), 12(e), and 12(f)). Nonstandard parameters make the gate circuit behave in nonstandard ways like in phantom limb pain, pain in demyelinating syndromes, breakthrough pain, and wind-down and wind-up pain. Readers can confer that parameters of Figure 12(b) allow phantom pain only in the case of no inputs to the gate (Figure 7(c)).

The versatility of pain responses obtained by the computational model is a consequence of the vertical symmetry of the proposed gate architecture (Figure 1(b)) in which all afferents to the circuit are excitatory. For the gate circuit, there is no way to differentiate a nociceptive or a sensory afferent. The only difference is that the spiking rate from mechanoreceptors is higher than from nociceptors due to the lower mechanoreceptors firing threshold and to the absence of a myelin sheath in nociceptors. When mechanoreceptor intensity (normalized spiking rate) is weak, as in demyelinating syndromes, the gate circuit is, some way, “deceived” and its dynamics become the same as if the sensory input was a nociceptive input.

**Figure 13:**
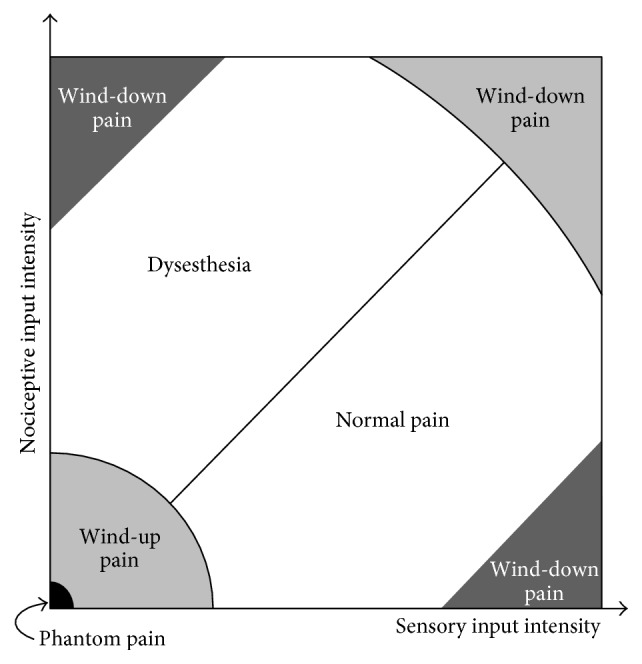
Different pain conditions for the different combinations of sensory and mechanoreceptor inputs according to the results of the gate circuit computer model. When the sensory input intensity (normalized firing rate) is higher than the nociceptive input intensity (below the diagonal), the circuit behaves in a “normal pain” mode. Above the diagonal, nociceptive input intensity is higher than the sensory input intensity. In this condition, the parameters of the gate circuit evolve so that pain is triggered in abnormal situations generating dysesthesia. When sensory and/or nociceptive inputs are very low, the gate circuit parameters evolve to produce wind-up pain. Phantom pain is included in this case as an extreme situation. Finally, when either nociceptive or mechanoreceptor stimulus is extremely high, wind-down pain is produced (see Figure 14).

The chart of Figure 13 represents an attempt to characterize the “standard” and “nonstandard”* modus operandi* of the gate circuit concerning the intensity of mechanoreceptor and nociceptive inputs. The sensory input intensity (the *x* coordinate) represents the normalized mechanoreceptor spiking rate measured at the mechanoreceptor axon terminal whereas the *y*-coordinate represents the normalized nociceptive spiking rate measured at the nociceptive terminal. The normalization is performed by dividing the spiking rate in a certain axon terminal by the highest possible spiking rate in any of the two axon terminals.

By exploring Figure 13, we can see that the diagonal is the place where sensory and nociceptive intensities are equal. Along the diagonal, and due to the symmetry of the circuit, it is not possible for the circuit to establish a difference between sensory and nociceptive inputs. From the point of view of neurons in the gate circuit, the two inputs are equivalent.

Below the diagonal of Figure 13, sensory inputs are more intense than nociceptive ones so that a standard type of stimulation is possible in this region.

Above the diagonal, sensory signals are weaker than nociceptive signals. Demyelinating syndromes like multiple scleroses and Guillain-Barré syndrome can reduce the rate of mechanoreceptor signals, thereby producing such situation. In these demyelinating syndromes, the network can misinterpret incoming signals so that the weaker mechanoreceptor input is treated like a nociceptive one, thereby generating a pain signal from CT neurons in a condition called dysesthesia.

We must emphasize that Figure 13 only shows two of the at least 8 initial parameters that influence the final set-point of the circuit in which parameters stabilize and a pain condition is established. Besides the two inputs' value, the other six parameters are the value of the four modifiable synaptic weights, *w*, and the value of the firing threshold, *t*, of the two neurons of the circuit.

**Figure 14:**
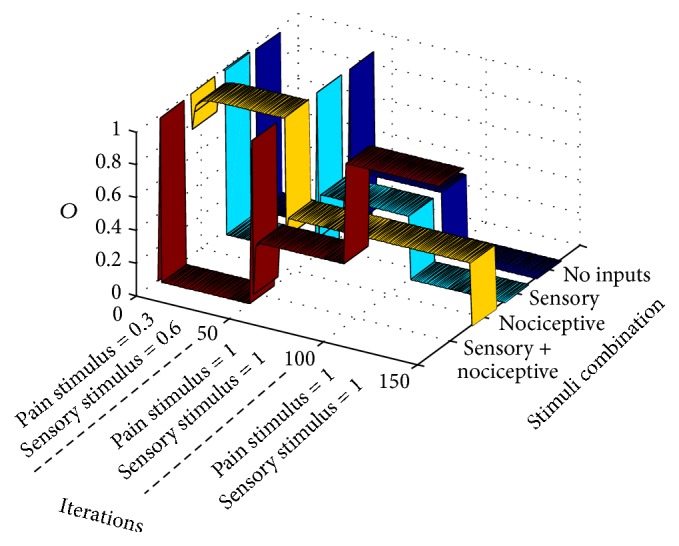
Modelling the hypothetical situation where, after a period of standard stimulation, gate circuit inputs are both very intense as from iteration 50 to iteration 100. After iteration 100, the four different combinations of nociceptive/sensory inputs are presented to the circuit. In the present case, as in Figures 10 and 11 cases, plasticity is blocked from iterations 100 to 150, in order to analyze the response of the circuit. As can be noticed, pain response is only intense when both sensory and nociceptive inputs are intense and are simultaneously applied. When only a nociceptive intense input is applied, the pain response is moderate. No pain response is obtained in the remaining cases.

Other nonstandard pain conditions are depicted in the chart: like wind-up pain that is produced, according to the computational model, when a continuous weak stimulation is delivered to a subject and is manifested by pain sensations in the intervals without stimuli similar to dysesthesia (see Section 3.2.5). Phantom pain is an extreme case of wind-up pain in which sensory and nociceptive inputs are zero (see Section 3.2.1). According to the model, wind-down pain takes place when either very intense sensory or nociceptive stimuli are delivered. One interesting case that was not tested in previous sections is the hypothetical situation in the right upper corner of the chart where intense nociceptive and sensory stimuli are delivered simultaneously and continuously. Although the gate usually mediates a wind-down phenomenon (as shown in Figure 14), unpredictable behaviors also take place because the diagonal is the main feature in this region of the chart.

Chart of Figure 13 is far from being final. Future research will surely contribute to improve this chart. In this paper, we trained the gate circuit with very specific combinations of sensory and nociceptive inputs. For the chart to be complete, a continuous sequence of sensory and nociceptive stimuli should be input to the gate. With each stimulus, little disturbances can be applied for gathering statistic measurements in order to study other aspects of gate circuit dynamics like stability and robustness.

This paper research shows that pain conditions are the result of a dynamic adjustment of circuit parameters in the presence of different type of stimulation that are external and/or derived from internal conditions like axon conductivity in demyelinating syndromes. We showed that the interplay between synaptic and intrinsic plasticity is necessary for the circuit to settle down in stability points that allow standard and nonstandard gate operations.

## 5. Conclusions

In this paper, we present a parsimonious computational model of the gate circuit that is able to account for a large number of different pain conditions. When compared with the Melzack and Wall gate circuit, our model only considers strictly excitatory afferents to the gate. However, the pain conditions modelled by this simpler architecture are numerous: it models normal gate functioning, phantom limb pain condition, wind-up and wind-down pain, breakthrough pain, and demyelinating syndromes like Guillain-Barré and multiple sclerosis. Two very simple equations allow the adaptation of synaptic weights (4) and firing thresholds (5) when different input patterns are delivered to the circuit. In the case of standard gate operation, modelled synaptic weights and firing thresholds values converge to very specific values independently of their starting values. The obtained parameters allow the same normal operation of the real gate circuit. When stimulation is not standard, due to external or internal factors, like demyelination syndromes, modelled synaptic weights and thresholds spontaneously settle down in stability points that give rise to precisely the same nonstandard pain conditions associated with the nonstandard type of stimulation. For example, intense constant stimuli produce pain reduction in the model like in real wind-down pain; weak constant sensorial stimuli produce growing pain in the model like in real wind-up pain; weakening of mechanoreceptor inputs produces abnormal pain (allodynia) in the model like in real demyelinating syndromes; and null inputs produce abnormal pain sensations (dysesthesia) like in phantom pain.

Our work opens a door in which computational models can be useful for treating pain syndromes: for relieving pain, it is possible to plan a strategy involving plasticity blockers or plasticity enhancers, together with stimulation schedules. This plan can be initially tested in a computer environment so that a personalized strategy can be planned depending on the subject's pain condition.

## Figures and Tables

**(a):** 

Nociceptive input	Mechanoreceptor input
0	0
0	1
1	0
1	1

**(b):** 

Nociceptive input	Mechanoreceptor input
0	0
0	0.6
**0.3**	0
**0.3**	0.6

**Table 2:** Stimulus intensity and firing probability of sensory/nociceptive receptors. For the qualitative purposes of our research, we elaborate a table that arbitrarily associate a verbal expression describing the intensity of a sensory/nociceptive stimulus with a firing probability interval.

Verbal expression for stimulus intensity	Firing probability intervals in sensory/nociceptive receptors
Very intense	(0.8, 1]
Intense	(0.6, 0.8]
Medium	(0.4, 0.6]
Weak	(0.25, 0.4]
Very weak	[0, 0.20]

**Table 3:** Output of CT neuron, *O*_2_, for each of the *I*_1_, *I*_2_ input pairs in the standard gate operation calculated from stability weights and thresholds represented in Figure 12(a): Net_1_ is obtained applying (2). In this case Net_1_ is calculated as Net_1_ = *w*_1_ *I*_1_ + *w*_3_ *I*_2_ for each pair. Neuron 1 output, *O*_1_, is equal to 1 when Net_1_ is higher than its threshold, *t*_1_. Net_2_ is calculated having into account neuron 1 inhibitory output: Net_2_ = *w*_2_ *I*_1_ + *w*_4_ *I*_2_ − *O*_1_. Finally the pain signal relayed to the brain from neuron 2 is triggered when Net_2_ > *t*_2_.

*I*_1_	*I*_2_	*w*_1_	*w*_3_	Net_1_	*t*_1_	*O*_1_	*w*_2_	*w*_4_	Net_2_	*t*_2_	*O*_2_
**0**	**0**	1	0.5	0	0.51	0	0	0.5	0	0.02	**0**
**0**	**0.3**	1	0.5	0.15	0.51	0	0	0.5	0.15	0.02	**1**
**0.6**	**0**	1	0.5	0.6	0.51	1	0	0.5	−1	0.02	**0**
**0.6**	**0.3**	1	0.5	0.75	0.51	1	0	0.5	−0.85	0.02	**0**
